# *In vivo* model to study the impact of genetic variation on clinical outcome of mastitis in uniparous dairy cows

**DOI:** 10.1186/s12917-020-2251-8

**Published:** 2020-01-31

**Authors:** L. Rohmeier, W. Petzl, M. Koy, T. Eickhoff, A. Hülsebusch, S. Jander, L. Macias, A. Heimes, S. Engelmann, M. Hoedemaker, H. M. Seyfert, C. Kühn, H. J. Schuberth, H. Zerbe, M. M. Meyerholz

**Affiliations:** 1 Clinic for Ruminants with Ambulatory Clinic and Herd Health Services, Center for Clinical Veterinary Medicine, Ludwig-Maximilians-University Munich, Sonnenstrasse 16, 85764 Oberschleissheim, Germany; 2 Clinic for Swine, Small Ruminants, Forensic Medicine and Ambulatory Service, University of Veterinary Medicine Hannover Foundation, Bischofsholer Damm 15, 30173 Hannover, Germany; 3 Immunology Unit, University of Veterinary Medicine Hannover Foundation, Bünteweg 2, 30559 Hannover, Germany; 4 Clinic for Poultry, University of Veterinary Medicine Hannover Foundation, Bünteweg, 17 30559 Hannover, Germany; 5 Leibniz Institute for Farm Animal Biology, Genome Biology, Wilhelm-Stahl-Allee 2, 18196 Dummerstorf, Germany; 6 Technical University Braunschweig, Institute for Microbiology, Inhoffenstrasse 7, 38124 Braunschweig, Germany; 7 Helmholtz Center for Infection Research, Microbial Proteomics, Inhoffenstrasse 7, 38124 Braunschweig, Germany; 8 Clinic for Cattle, University of Veterinary Medicine Hannover Foundation, Bischofsholer Damm 15, 30173 Hannover, Germany; 9 Agricultural and Environmental Faculty, University Rostock, Justus-von-Liebig-Weg 6, 18059 Rostock, Germany

**Keywords:** *Staphylococcus aureus*, *Escherichia coli*, BTA18, Genetic selection, Haplotype, Intramammary infection model, Somatic cell count, Mastitis

## Abstract

**Background:**

In dairy herds, mastitis causes detrimental economic losses. Genetic selection offers a sustainable tool to select animals with reduced susceptibility towards postpartum diseases. Studying underlying mechanisms is important to assess the physiological processes that cause differences between selected haplotypes. Therefore, the objective of this study was to establish an *in vivo* infection model to study the impact of selecting for alternative paternal haplotypes in a particular genomic region on cattle chromosome 18 for mastitis susceptibility under defined conditions in uniparous dairy cows.

**Results:**

At the start of pathogen challenge, no significant differences between the favorable (Q) and unfavorable (q) haplotypes were detected. Intramammary infection (IMI) with *Staphylococcus aureus 1027* (*S. aureus*, *n* = 24, 96 h) or *Escherichia coli 1303* (*E. coli*, *n* = 12, 24 h) was successfully induced in all uniparous cows. This finding was confirmed by clinical signs of mastitis and repeated recovery of the respective pathogen from milk samples of challenged quarters in each animal. After *S. aureus* challenge, Q-uniparous cows showed lower somatic cell counts 24 h and 36 h after challenge (*P* < 0.05), lower bacterial shedding in milk 12 h after challenge (*P* < 0.01) and a minor decrease in total milk yield 12 h and 24 h after challenge (*P* < 0.01) compared to q-uniparous cows.

**Conclusion:**

An *in vivo* infection model to study the impact of genetic selection for mastitis susceptibility under defined conditions in uniparous dairy cows was successfully established and revealed significant differences between the two genetically selected haplotype groups. This result might explain their differences in susceptibility towards IMI. These clinical findings form the basis for further in-depth molecular analysis to clarify the underlying genetic mechanisms for mastitis resistance.

## Background

For decades, mastitis has caused large-scale economic losses worldwide in dairy farming due to treatment costs, discarded milk, reduced milk yield and increased culling rates [1–6]. A recent study from Canada estimated costs on typical dairy farms to be 662 Canadian Dollars per milking cow per year, in which nearly half of the costs were associated with subclinical mastitis [7]. Additionally, indirect costs arise due to reduced fertility of cows suffering from clinical or subclinical mastitis [8–10]. Clinical mastitis (CM) is defined as intramammary infection (IMI) with clinical symptoms, such as altered milk secretion, local (pain, swelling) or systemic signs of inflammation (fever, disturbed general condition). IMI with *Escherichia coli* (*E. coli*) frequently causes CM, which can severely affect the well-being of the animal but often results in transient IMI with a comparably high self-cure rate [11–15]. In comparison, subclinical mastitis (SCM) includes IMI without clinical symptoms but an increased somatic cell count (SCC) in milk, decreased milk yield and reduced milk quality. *Staphylococcus aureus* (*S. aureus*) is one major pathogen causing SCM or mild cases of CM in dairy cows [16]. Due to intermittent shedding, *S. aureus* is difficult to detect, and treatment of affected animals is often futile, since *S. aureus* IMI tends to persist within the udder and causes chronic cases of SCM [1, 6, 17]. In the dairy industry, CM and SCM are the major reasons for antimicrobial usage [18, 19]. Additionally, cows with CM or SCM are prone to suffer from other diseases [8, 20, 21]. Several studies have reported correlations between different reproductive and metabolic disorders and respective management strategies to be the key factor for improvement in this area [15, 22–24]. This improvement aims not only to reduce the antimicrobial usage in dairy cows but also to meet the requirements of well-informed and demanding consumers of dairy products. Irrespective of economic aspects, mastitis and its associated implications have detrimental effects on animal welfare [25]. Genetic selection offers a sustainable tool to select animals with decreased susceptibility towards postpartum diseases. Several groups have reported promising associations between *Bos taurus* autosome 18 (BTA 18) and performance traits [26–29]. Our own studies revealed differing immune competence of primary mammary epithelial cells (MEC) originating from two BTA 18 haplotypes: half sib uniparous cows inheriting an alternative haplotype of a confirmed quantitative trait locus (QTL) for somatic cell score (SCS) in the telomeric region of BTA 18 showed different somatic cell scores* in vivo* [30]. The MECs of these uniparous cows differed in their expression profiles after pathogen challenge *in vitro* [31, 32]. These findings indicate a reduced susceptibility towards intramammary infections in uniparous cows inheriting the favorable QTL allele. Another study recently showed that in addition to selection for disease susceptibility, host infectivity should be considered an important aspect in efficiently reducing diseases in cattle [33]. Studying the underlying mechanisms is important to explore the physiological processes, which cause the reported differences between the haplotypes to carve out and benefit from positive implications and to be aware of negative implications of applied selection strategies. Numerous experimental *in vivo* mastitis models have been established by various researchers over the last several decades, as recently reviewed by Petzl et al. (2018) [34]. However, to the best of our knowledge, no* in vivo* mastitis model comparing differing BTA 18 haplotype uniparous cows has been performed to date. Therefore, the objective of this project was to establish an *in vivo* infection model to study the impact of genetic selection for mastitis resistance under defined conditions in uniparous dairy cows. During the selection process of the BTA 18 haplotypes, SCC served as a target phenotype for mastitis incidence and udder health. The severity and resolution of mastitis is known to be heavily influenced by the species of the infecting pathogen [35], and it was shown that Gram-negative pathogens trigger different immune reactions in the host compared to Gram-positive pathogens [16]. To address the pathogen-specific clinical outcome of mastitis, *E. coli* served as a surrogate pathogen for acute CM and *S. aureus* as a surrogate pathogen typically causing SCM or mild CM in dairy cows. The suitability of both strains to serve as typical pathogens has recently been demonstrated [16].

## Results

### Successful establishment of an* in vivo* infection model

No major pathogens were detected in the last bacteriological examinations of milk samples obtained from each uniparous cow before the start of the challenge experiment. At the start of the experimental challenge, the animals were free of withdrawal periods, and none of the animals showed signs of systemic diseases.

**Fig. 1:**
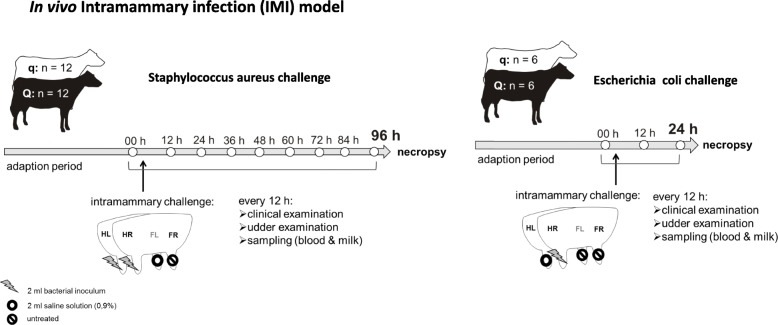
Graphical illustration of the *in vivo* intramammary infection (IMI) model. Animals selected for paternal BTA 18 haplotypes favorable (Q, *n* = 18) or unfavorable (q, *n* = 18) for somatic cell count received intramammary challenge with *Staphylococcus aureus1027* (*n* = 24) or *Escherichia coli1303* (*n* = 12) for 96 or 24 h, respectively. During *Staphylococcus aureus* challenge, inoculation was performed in the hind left (HL) and hind right (HR) quarters, and the front left (FL) quarter served as a negative control inoculated with saline solution, while the front right (FR) quarter was not treated. During *Escherichia coli* challenge, inoculation was performed with HR, saline solution was administered HL and front quarters were untreated. Clinical examination, udder examination and blood and milk sampling were performed every 12 h. The graphical illustration of the cow and the udder has been designed by Wolfram Petzl

**Fig. 2:**
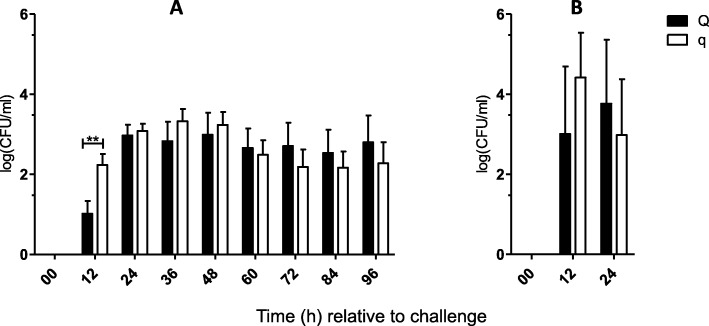
Graphical illustration of colony forming units isolated from Q-/q-uniparous cows after intramammary challenge. Colony forming units logarithmized to the base 10 per milliliter (log (CFU/ml)) of bacteria isolated from sterile milk samples of infected udder quarters after intramammary challenge with (**a**) *Staphylococcus aureus* (Q: *n* = 12 versus q: *n* = 12) and (**b**) *Escherichia coli* (Q: *n* = 6 versus q: *n* = 6) is shown. The first sample was taken before intramammary challenge and defined as 0 h relative to challenge. Afterwards, quarter milk samples were taken every 12 h. Data are presented as the mean and standard error of the mean (**a**) and as the median and interquartile range (**b**). Differences between uniparous cows selected for favorable (Q) and unfavorable (q) haplotypes are indicated with * if *P* < 0.05 and with ** if *P* < 0.01. Significant differences within the haplotype groups over time relative to challenge are not shown

**Fig. 3:**
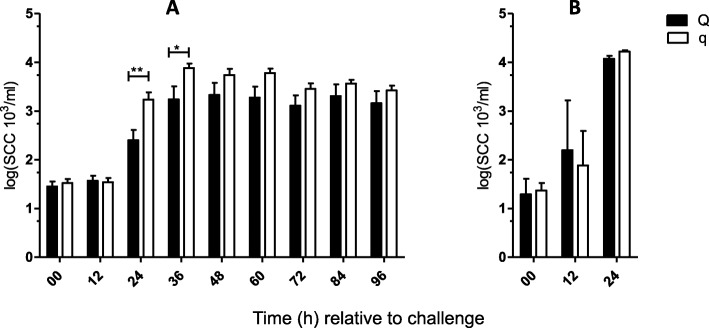
Graphical illustration of somatic cell count from Q-/q-uniparous cows after intramammary challenge. Somatic cell count (SCC) logarithmized to the base 10 in ml (log SCC 10^3^/ml) determined in milk sampled under sterile conditions from infected udder quarters after intramammary challenge with (**a**) *Staphylococcus aureus* (Q: *n* = 12 versus q: *n* = 12) and (**b**) *Escherichia coli* (Q: *n* = 6 versus q: *n* = 6) is shown. The first sample was taken before intramammary challenge and defined as 0 h relative to challenge. Afterwards, quarter milk samples were taken every 12 h. Data are presented as the mean and standard error of the mean (**a**) as the median and interquartile range (**b**). Differences between uniparous cows selected for favorable (Q) and unfavorable (q) haplotypes are indicated with * if *P* < 0.05 and with ** if *P* < 0.01. Significant differences within the haplotype groups over time relative to challenge are not shown

**Fig. 4:**
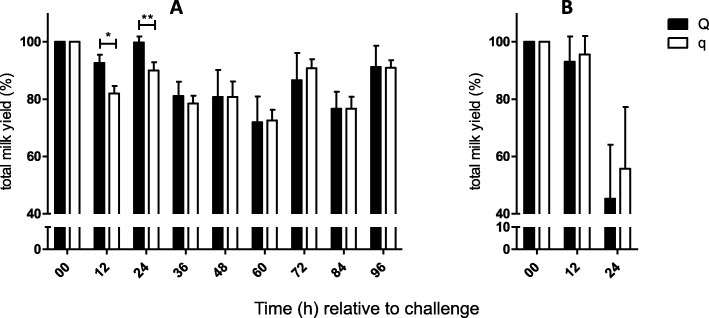
Graphical illustration of total milk yield from Q-/q-uniparous cows after intramammary challenge. Total milk yield in percent (%) relative to total milk yield at the start of the challenge (0 h) of uniparous cows after intramammary challenge with (**a**) *Staphylococcus aureus* (Q: *n* = 12 versus q: *n* = 12) and (**b**) *Escherichia coli* (Q: *n* = 6 versus q: *n* = 6) is shown. Milking was performed every 12 h after challenge, and total milk yield was determined. Data are presented as the mean and standard error of the mean (**a**) as the median and interquartile range (**b**). Differences between uniparous cows selected for favorable (Q) and unfavorable (q) haplotypes are indicated with * if *P* < 0.05 and with ** if *P* < 0.01. Significant differences within the haplotype groups over time relative to challenge are not shown

Intramammary infection with *S. aureus* (*n* = 24, 96 h) or *E. coli* (*n* = 12, 24 h) was induced in all uniparous cows, and samples were obtained every 12 h after IMI, as illustrated in Fig. 1. The success of intramammary infection was confirmed by clinical signs of mastitis: changes in milk secretion and udder firmness were observed after challenge with both pathogens (Tables 1 and 2). Repeated recovery of the respective pathogen from milk samples in each cow also served to confirm the success of the intramammary infection. Quantification of bacteria was performed via plate count of colony forming units (CFU) per ml (Fig. 2). A significant increase in SCC and a decrease in total milk yield were observed after challenge with both pathogens (Figs. 3 and 4). The first signs of mastitis were detected 24 h after *S. aureus* challenge. As expected, the onset of local changes after intramammary challenge with *E. coli* was earlier (12 h) and higher in severity compared to animals challenged with *S. aureus* (Tables 1 and 2). The inner body temperature was measured every three minutes via an intravaginal temperature logger. Nearly all uniparous cows belonging to the *S. aureus* group (23 out of 24 animals) developed fever, defined as inner body temperature ≥ 39.5°Celsius (C), during the 96-h trial. All uniparous cows belonging to the *E. coli* group developed fever during the 24 h trial. The maximum body temperature was significantly higher in the *E. coli* group (41.8 °C ± 0.2) compared to the *S. aureus* group (40.9 °C ± 0.2; *P* = 0.002), indicating a pathogen-specific host response towards intramammary challenge.

**Table 1:** Local effects on milk after intramammary challenge of Q-/q-uniparous cows with *S. aureus* or *E. coli*

Udder secretion score^a^	*Time (h) relative to S. aureus challenge*																		*Time (h) relative to E. coli challenge*					
	*0*		*12*		*24*		*36*		*48*		*60*		*72*		*84*		*96*		*0*		*12*		*24*	
	*Q*	*q*	*Q*	*q*	*Q*	*q*	*Q*	*q*	*Q*	*q*	*Q*	*q*	*Q*	*q*	*Q*	*q*	*Q*	*q*	*Q*	*q*	*Q*	*q*	*Q*	*q*
*Normal milk*	12	12	12	12	11	7	8	2	5	2	6	1	5	2	5	2	2	2	6	6	6	6	1	1
*Watery milk, no clots*					1	2	1	4				1	2	1			1							
*Milk, few small clots*							2	3	3	4	1	3	1	3	2	4	3	3					2	1
*Milk, several clots*						2	1	2	2	3	2	4	1	2	4	3	3	4						1
*Milk, many clots*						1				2			2	1	1	1	2	2						
*Serous milk, mainly clots*									1	1	1	3		2		2		1						
*Serum-like secretion*								1	1		2		1	1			1						3	3

^*a*^*udder secretion score represents signs of inflammation in milk secretion. Animals with divergent haplotypes (Q/q) received intramammary challenge with S. aureus (24 animals (Q: n = 12* versus *q: n = 12), 2 udder quarters) or E. oli (12 animals (Q: n = 6* versus *q: n = 6), 1 udder quarter). For S. aureus-challenged animals, a mean score is given for two challenged udder quarters*

**Table 2:** Udder parenchyma firmness after intramammary challenge of Q-/q-uniparous cows with *S. aureus* or *E. coli*

Udder palpatory score^a^	*Time (h) relative to S. aureus challenge*																		*Time (h) relative to E. coli challenge*					
	*0*		*12*		*24*		*36*		*48*		*60*		*72*		*84*		*96*		*0*		*12*		*24*	
	*Q*	*q*	*Q*	*q*	*Q*	*q*	*Q*	*q*	*Q*	*q*	*Q*	*q*	*Q*	*q*	*Q*	*q*	*Q*	*q*	*Q*	*q*	*Q*	*q*	*Q*	*q*
*Soft*	1		1																					
*Soft, small knots*	8	9	5	7	2	4	3			1		2	1	1		1		1	3	5	2			
*Coarse, small knots*	3	3	6	5	9	7	6	10	9	9	8	7	7	8	8	8	8	7	3	1	1	3	1	2
*Coarse, large knots*					1	1	3	2	2	1	2	2	2	2	3	2	3	3			1		2	
*Firm, no subcutis edema*										1	1	1	1		1						1	1	1	2
*Quarter swelling*									1		1		1	1		1	1	1			1	2	2	2

^*a*^*udder palpatory score represents signs of inflammation (firmness) of udder parenchyma. Animals with divergent haplotypes (Q/q) received intramammary challenge with S. aureus (24 animals (Q: n = 12* versus *q: n = 12), 2 udder quarters) or E. coli (12 animals (Q: n = 6* versus *q: n = 6), 1 udder quarter). For S. aureus-challenged animals, a mean score is given for two challenged udder quarters*

### Comparable systemic effects after pathogen challenge in Q and q

To evaluate the severity of the induced mastitis and to detect differences between the divergent haplotypes, the general health condition of the uniparous cows was monitored via such parameters as heart rate, inner body temperature, filling and activity of the rumen and feed intake and scored according to a general health condition scoring scheme (Score 0–9, Additional file 1: Table S1) modified based on Petzl et al. (2012) [36]. In the *S. aureus* group, as well as in the *E. coli* group, no significant differences concerning general health condition between the divergent haplotypes during the experimental setup were found (Additional file 2: Table S2 and Additional file 3: Table S3). Maximum general health condition score within the 96 h after intramammary challenge with *S. aureus* or within 12 h after intramammary challenge with *E. coli* did not differ between Q- and q-uniparous cows (*S. aureus*-group: Q: 2.88 ± 0.44 versus q: 2.92 ± 0.40; *P* = 0.95; *E. coli*-group Q: 2.5 ± 2.88 versus q: 1.75 ± 2.25; *P* = 0.62). Furthermore, the time point of maximum general health condition score did not differ between the two groups: (*S. aureus*-group Q: 42 h *p. inf.* ± 33 versus q: 36 h *p. inf.* ± 21; *P* = 0.78.; *E. coli*-group Q: 12 h ± 6 versus q: 6 h ± 12; *P* = 0.62).

Intravaginal temperature during the challenge did not differ between Q- and q-uniparous cows, regardless of maximum body temperature (*S. aureus* group: Q: 41.0 °C ± 0.3 vs. q: 40.8 °C ± 0.2; *E. coli* group: Q: 41.8 °C vs. q: 42.1 °C; *P* ≥ 0.1) or concerning the time point of the maximum inner body temperature after challenge (*S. aureus* group: Q: 33.4 h ± 13.5 vs. q: 30.1 h ± 13.1; *E. coli* group: Q: 14.3 h vs. q: 15.7 h; *P* > 0.1). During the challenge period, no severe general depression, recumbency, gangrenous mastitis or rectal temperature > 42.5°C were observed such that exclusion of animals from the experiment or termination of the experiment was not necessary.

### No differences in local clinical signs of mastitis between haplotypes

Local signs of CM were examined every 12 h using a milk secretion and an udder palpation scoring system not only to prove the success and evaluate the extent of the experimentally induced mastitis as described above but also to compare these local effects between the divergent haplotype groups. No differences between the Q- and the q-haplotype were detected in this regard, either within the *S. aureus* or within the *E. coli* group (Tables 1 and 2).

### Lower *S. aureus* shedding in milk of Q compared to q relative to challenge

Bacterial recovery of the inoculated pathogen in the milk served as a criterion to prove a successfully induced IMI. Q-uniparous cows infected with *S. aureus* showed significantly lower bacterial shedding in milk samples of infected quarters 12 h after challenge (Fig. 2) compared to q-uniparous cows. No significant differences between Q- and q-uniparous cows concerning bacterial shedding were detected in the *E. coli* group (Fig. 2).

### Lower SCC in Q compared to q after intramammary *S. aureus* challenge

The SCC applies as the main parameter to evaluate udder health because it indicates the inflammatory response during an IMI. At the time point before the intramammary challenge (0 h), all uniparous cows included in this study showed mean low SCC, and no significant differences were detected between the divergent haplotypes (Q: 42.2 * 10^3^/ml ± 10.1 vs. q: 58.5 * 10^3^/ml ± 12.5; P > 0.1; Fig. 3). A significant increase in SCC was detected in the infected quarters of all 24 uniparous cows from the *S. aureus* group 24 h after intramammary challenge. Comparison of the haplotypes revealed significant differences concerning SCC during the course of the experiment: 24 and 36 h after challenge, Q-uniparous cows showed lower SCC levels in milk samples from infected quarters compared to q-uniparous cows (Fig. 3). The SCC of milk samples from noninfected udder quarters did not differ between Q- and q-uniparous cows (*data not shown*). In the *E. coli* group, a significant increase of SCC in the milk of the infected quarter was detected earlier compared to the *S. aureus* group already at 12 h after challenge, but no differences between the divergent haplotypes have been found (Fig. 3).

### Minor drop of total milk yield in Q compared to q after intramammary *S. aureus* challenge

Total milk yield declined in all uniparous cows after intramammary challenge with either *S. aureus* or *E. coli* (maximal decline ~ 35% and ~ 50%, respectively; Fig. 4). In the *S. aureus* group, the decline of total milk yield was ~ 10% less pronounced in Q- compared to q-uniparous cows: the total milk yield in percent 12 h and 24 h after challenge relative to that at the beginning of the challenge was higher, and 12 h and 24 h after challenge, total milk yield in percent relative to that at the start of the challenge was higher in Q- compared to q-uniparous cows (12 h after challenge Q: 92.7% ± 2.8 vs. q: 82.0% ± 2.2; *P* < 0.05; 24 h after challenge Q: 99.8 ± 2.0 vs. q: 90.1 ± 2.8; *P* < 0.01; Fig. 4). In contrast, Q- and q-uniparous cows did not differ concerning the reduction in total milk yield after intramammary challenge with *E. coli*.

## Discussion

The objective of this study was to establish an *in vivo* infection model to study the impact of genetic selection for mastitis susceptibility under defined conditions in uniparous dairy cows. Thirty-six Holstein Friesian uniparous cows selected for favorable (Q) and unfavorable (q) paternal BTA 18-haplotypes for SCC were included in this study. SCC served as a surrogate trait for mastitis susceptibility, indicating low (Q) or high (q) mastitis susceptibility. At the start of the experiment, no significant differences regarding udder-specific parameters were found between the two haplotypes, although Q-uniparous cows had shown a lower incidence of metritis, lower blood concentrations of betahydroxbutyrate compared to q-uniparous cows, numerical lower incidence of CM and SCM in the postpartum period and significant differences in SCC as recently published [37–39]. Accurate surveillance before and after calving was essential to prepare and synchronize the two haplotype groups, despite differing periparturient performance. The experiment was conducted as planned in all uniparous cows, and none of the animals had to be excluded from the study based on defined exclusion criteria. After intramammary challenge with *E. coli* or *S. aureus*, all animals developed IMI and displayed clinical signs of mastitis in a pathogen-specific manner, and re-isolation of the respective pathogens was successful in all cases. A significant increase in the SCC and decrease in milk yield was assessed after *S. aureus* challenge, as well as after *E. coli* challenge. This finding complies with results of previous studies, which compared pathogen- and time-dependent variability of the innate immune response in dairy cows challenged with *S. aureus* or *E. coli* [40, 41]. It can be generalized from the results that via thorough standardization of the animals and their environment, establishment of an intramammary infection model to study the influence of the respective haplotype was achieved. Synchronization of Q- and q-uniparous cows was achieved to such an extent that genetically determined differences were not blurred by environmental effects.

In the present study, the two BTA 18 haplotype groups showed initial differences during their clinical response towards experimental IMI, but these differences were limited to *S. aureus* IMI. Significantly lower SCC in Q- compared to q-uniparous cows 24 h and 36 h after challenge with *S. aureus* and significantly lower bacterial load in milk samples 12 h after challenge may suggest differing capacities of antimicrobial reaction patterns between the two haplotype groups. The less prominent decrease in total milk yield 12 h and 24 h after challenge with *S. aureus* in Q- compared to q-uniparous cows completes this picture of less intense reaction towards intramammary challenge in Q-uniparous cows. This result, in turn, indicates that Q-haplotype uniparous cows are more resilient against IMI than those featuring the q-haplotype. These findings prove that the genetic selection for chromosome BTA 18 haplotypes performed in this study has an impact on experimentally induced mastitis. It would be eligible to further explore the way of genetic selection using indirect parameters as well as results of resistance to infection to define robust biomarkers in the future. It was unexpected that differences in the clinical response between haplotypes during *S. aureus* IMI could clinically be discriminated only in the initial phase. One decade ago, Rupp et al. (2009) published their study concerning an animal model with two divergent groups of ewes that had been selected for reduced susceptibility towards IMI based on SCS [42]. The selection criteria included extreme breeding values of the respective rams, but no genotyping was applied. The results indicated that ewes from the ‘high SCS line’ revealed sustained better capacities to eliminate IMI after parturition and during lactation. However, these ewes were only confronted with naturally occurring IMI, and no controlled experimental challenge model was established to carefully scrutinize the genetically determined differential resilience against IMI. To the best of our knowledge, no comparable studies have been published to date reporting on experimentally induced IMI to compare the impact of BTA 18 haplotypes on the resolution and outcome of mastitis in dairy cows.

After IMI with *E. coli,* no differences were found between Q- and q-uniparous cows based on our diagnostic parameters. This lack of differences might be caused by different reasons. First, the virulence of the Gram-negative *E. coli* pathogen was higher than that of the *S. aureus* pathogen, as evidenced by the finding that bacterial counts in milk 12 h after IMI increased by more than orders of magnitude. This finding caused a stronger assault than *S. aureus* infection and elicited a strong host reaction that might have overridden the effectiveness of the defense mechanisms influenced by the genetic selection, as applied in this study. Second, the host immune defense against mammary infection with Gram-negative (e.g., *E. coli*) infection is governed and determined by mammary epithelial cells (MEC), while this dominant cell type of the lactating udder does only play a minor role in defending against Gram-positive mammary pathogens, such as *S. aureus* or *Streptococcus uberis* [16, 43]. Hence, those immune mechanisms determined by the BTA 18 haplotype having been selected for in this study might not reside in MEC but rather in other immune-relevant cell types. This conclusion agrees notably well with those of Bonnefont et al. 2012 [44], who analyzed MEC from genetically selected ewes of different resilience against mastitis.

IMI models with intramammary application of *E. coli* that were previously performed within our work group were limited to 24 h [40, 45]. In these studies, pathogen-specific reaction patterns were demonstrated, and maximum inner body temperature and changes in milk secretion were detected approximately 12 h to 14 h after challenge. Due to ethical reasons and to preserve comparability of the results with previous studies, *E. coli* IMI was limited to 24 h in the present study, as well. However, it was unexpected that uniparous cows in this challenge experiment showed macroscopic changes in milk secretion and udder firmness not before 24 h after challenge. One explanation for this delayed reaction of the mammary tissue might be that the animals were early-lactating animals compared to mid-lactating animals, which had been used in previous studies. Vangroenweghe et al. (2004) demonstrated that early lactating primiparous cows showed moderate clinical symptoms towards IMI with *E. coli* [13], and Van Werven et al. (1997) showed a significant effect of parity on the severity of clinical mastitis induced via *E. coli* [46]. It is further known that the clinical course of IMI induced via *E. coli* might be quite severe, but the infection is self-limited, and a high self-cure rate can be observed. Hence, it can only be speculated whether further sampling for a longer period would have revealed more striking differences between the two haplotypes concerning bacteriological and clinical cure of *E. coli* IMI.

Another unexpected result was the frequent observation of fever during *S. aureus* IMI. The reason for this finding might be the close monitoring of the inner body temperature via the intravaginal device, recording data every three minutes. In previous studies, rectal temperature was only measured every 6–12 h [40], meaning that potential peaks in between might have been missed.

In their review, Schukken et al. (2011) summarized that long-term self-cure in *S. aureus* IMI is possible and that persistence of the bacteria within the udder varies from individual to individual [35]. The course of infection/inflammation in this study could not be monitored longer than 96 h due to limitations within the experimental setup; thus, the resolution or persistence of the *S. aureus* infection in the long term has not been determined. Because ewes from the ‘high SCS line’ were more susceptible to natural IMI with clinical symptoms [42], it would have been revealing to compare the efficiency of the two haplotypes in eliminating the bacteria from the infected mammary quarter, but this aspect was not within the scope of the present study.

In cattle, recently, several association studies revealed consistent results concerning BTA 18 and genetic modulation of functional traits [26–29]. Data from our work group obtained from the postpartum period of the divergent haplotype uniparous cows [37] and from a parallel long-term trial [38] indicate that although SCC served as a target criterion during the selection process, the metabolic adaption capacity of the uniparous cows seems to play a fundamental role in paving the way for adequate immune response patterns towards immunological challenges.

As reviewed by Petzl et al. (2018) [34], several *in vitro* and *in vivo* studies addressed pathophysiological processes involved in IMI and mastitis. The standardization of intramammary challenge models has been widely improved in recent years. In the present study, the aspect of divergent BTA 18 haplotypes of uniparous cows was integrated into an intramammary challenge model for the first time.

In summary, the requirements of intramammary challenge models to mimic CM or SCM have changed over the last several years, since new methods in animal breeding and transcriptomic profiling have become more complex. The established model sets new standards and can be used as a tool to assess molecular changes of the metabolome, proteome and transcriptome of the respective animals by novel techniques. During the next several years, admission, storage and management of big data from the dairy cow stables and associated machine learning represent one of the future challenges in the dairy industry to address economic feasibility, health status and welfare of the respective animals. Well-established animal models can build the basis for interpreting the relevant data for controlled advances in this field.

## Conclusions

An *in vivo* infection model to study the impact of specific genetic selection for mastitis susceptibility under defined conditions in uniparous dairy cows was successfully established in this study. Significant differences between the two genetically selected haplotypes focused on SCC and bacterial shedding, which might explain the differing susceptibility towards mastitis. These findings must be supplemented with further data from studies with regard to haplotype-dependent susceptibility towards natural infections and monitoring of subsequent lactations to clarify both the economic feasibility of that genetic selection scheme and the underlying immune mechanisms. The present challenge model is applicable for studying differences between groups of cows embedded in holistic approaches.

## Methods

### Animals

The objective of the study was to establish an *in vivo *infection model to study the impact of genetic selection for mastitis susceptibility under defined conditions in uniparous dairy cows.

The experiment included 36 Holstein Friesian uniparous cows that were genetically selected for favorable (Q) and unfavorable (q) paternal chromosome-18-haplotypes for somatic cell count (SCC), as previously described [37–39]. The SCC served as a surrogate trait for mastitis susceptibility; therefore, low SCC was assumed to represent low mastitis susceptibility (Q), and high SCC was assumed to represent high mastitis susceptibility (q). The trial was conducted between January and September 2016 under the approval of the Lower Saxony Federal State Office for Consumer Protection and Food Safety (reference number 33.12–42,502–04-15/2024; approval date: December 15th, 2015).

All uniparous cows were purchased from conventional private dairy farms across Germany and housed in individual pens at the Clinic for Cattle, University of Veterinary Medicine, Hannover, from at least four weeks before the calculated calving date until sacrifice after the intramammary challenge. The uniparous cows received constant veterinary care, including daily general examination, measurement of rectal temperature twice daily, calving management and treatments according to standard veterinary practice in case of diseases. After calving, detailed udder health monitoring was performed on a weekly basis, including udder palpation, macroscopic evaluation of milk secretion, cow-side California Mastitis Test (CMT) and quarter milk sampling for milk ingredients, SCC and microbiological examination.

Pens were cleaned twice daily and provided with fresh straw. The animals received a performance-adjusted component ration (dry-off, transition period, lactation period; concentrate adjusted daily according to milk yield). Water was given ad libitum. On days 1–6 postpartum (p.p.) all uniparous cows received 2500 mg enrofloxacin (Enrotron®100) per day to create comparable conditions between the two groups with regard to antibiotic treatment. The overall aim of this systematic antibiotic treatment within the experimental setup was to strictly synchronize the two haplotypes for maximal standardization of the experimental model including prevention of natural IMI before the start of the experiment. In case of disease, the treatment was prolonged.

Criteria for exclusion of animals from the experiment to create reliable and precise data and for ethical reasons were defined. Intramammary challenge was not conducted if (1) major pathogens have been detected in quarter milk samples at the last sampling before the start of the experiment, (2) treatment of occurring diseases had not been finished within one week before the start of the experiment, (3) withdrawal periods of applied pharmaceuticals had not expired at the start of the experiment or if (4) the respective animal showed clinical signs of a systemic disease before the start of the intramammary challenge. Furthermore, rectal temperature > 42.5 °C, general depression and recumbency, as well as gangrenous mastitis, were criteria for discontinuing the experiment.

### Intramammary infection model

The intramammary challenge experiment started on day 36 ± 3 p.p. The order in which the animals entered the experimental setup depended on the individual calving date. The uniparous cows were challenged intracisternally with either *S. aureus* (Q *n* = 12 vs. q n = 12) or *E. coli* (Q *n* = 6 vs. q n = 6). The time point before administration of the bacteria was defined as 0 h.

Intramammary *S. aureus* challenge was applied to the left and right hindquarters after cleaning and disinfection of the teats with 70% ethanol. The inoculum stock was diluted with sterile pyrogen-free 0.9% saline solution to a challenge dose of 10,000 CFU *S. aureus1027* /2 ml. The inoculum was instilled into the teat canal with a sterile syringe and a teat cannula. Afterwards, the teat canal was kept close with two fingers, and the udder was massaged for 30 s to ensure distribution of the respective pathogen. The front left quarter received 2 ml sterile pyrogen-free 0.9% saline solution, and the front right quarter remained untouched. Both front quarters served as control quarters. This protocol was followed for all uniparous cows within the *S. aureus* group with one exception: one cow had suffered from CM in the left hind quarter in the postpartum period, which had been treated and cured before the challenge, but to avoid an influence on the local intramammary reactivity, the pathogen was applied into the front right instead of the hind left quarter. The infection trial lasted 96 h after intramammary challenge with *S. aureus*.

Intramammary *E. coli* challenge (500 CFU *E. coli1303* / 2 ml) was applied into the hind right quarter. The front right and front left quarter were left untreated, and the hind left quarter received 2 ml sterile pyrogen-free 0.9% saline solution. The inoculum was instilled in the same manner as in the *S. aureus* group. The infection trial lasted 24 h after intramammary challenge with *E. coli*. This protocol was followed for all uniparous cows within the *E. coli* group with one exception: due to stenosis in the hind right and front left udder quarters, the pathogen was applied into the hind left quarter of this cow.

At the end of the experiment, the uniparous cows were killed with a captive bolt gun and exsanguination immediately followed by necropsy and tissue sampling for further investigations (Fig. 1).

### Pathogens for intramammary challenge

The applied strains of *S. aureus1027* and *E. coli1303* are field isolates from cases of subclinical and clinical mastitis, respectively. Genomic and proteomic characteristics of *S. aureus1027,* including common virulence markers and virulence gene expression, have been examined [47], and the genome sequence of *E. coli1303* has been published [48]. The strains were stocked in a cryobank system at − 80 °C. To create a stock solution for comparable intramammary challenge doses, *E. coli* was cultured on violet red bile agar (VRB), and *S. aureus* was cultured on columbia sheep blood agar (CSB) and incubated (24 h, 37 °C). Afterwards, one colony per bacterial strain was applied into a tube with 10 ml brain heart infusion broth (BHI) and subsequently incubated (6 h, 37 °C). Of this solution, 100 μl was applied into 9.9 ml tryptic soy broth (TSB). After 18 h of incubation, the inoculum was prepared to perform serial dilutions. These serial dilutions were plated on VRB (*E. coli*) or CSB (*S. aureus*) and incubated for 24 h to determine the counts of colony forming units per ml (CFU/ml) in the inoculum. The prepared inoculum was aliquoted and stored at − 80 °C.

### Monitoring and sampling

To monitor local and systemic signs of mastitis, all uniparous cows were examined immediately before the challenge (0 h) and every 12 h after challenge with regards to their general health and signs of inflammation in the udder and milk secretion. To evaluate the general health status, such parameters as heart rate, respiratory frequency, rectal body temperature, feed intake, rumen activity and content were recorded. Additionally, the inner body temperature was measured over the entire experimental challenge every three minutes via a temperature logger (HOBO U12 Stainless Temperature Data Logger, Onset Computer Corporation, Bourne; USA) attached to an intravaginal plastic device (EAZI-BEED CIDR-blank, Zoetis, USA) containing no progesterone. Due to data loss because of rejection of the intravaginal device in one animal, statistical analysis of the inner body temperature within the *E. coli* group could only be performed for *n* = 11 uniparous cows (Q: *n* = 6, q *n* = 5).

For assessing udder health before and during the challenge, all udder quarters were examined for signs of inflammation such as swelling, redness, pain or increased udder surface temperature as well as for the evaluation of milk secretion according to Table 1 and Table 2. Sterile quarter milk sampling was performed for bacterial examination (including colony counting) every 12 h before milking of the uniparous cows. These samples were stored on ice until further processing in the laboratory (see below). The milking procedure was conducted with a special quarter milker (WestafliaSurge, Bönen, Germany) to determine the exact amount of milk per quarter and to take quarter milk samples for the determination of SCC, pH and milk contents. Respective milk samples were preserved with bronopol for further analysis at Milchwirtschaftlicher Kontrollverband Mittelweser e.V. (Rehburg-Loccum, Germany) using the MilkoScan FT Plus (FOSS, Hilleroed, Denmark).

### Bacterial recovery from milk

Bacterial recovery was assessed via sterile sampling of quarter milk samples. Each sample was streaked onto three different agar plates (CSB, VRB, Edwards-Agar) and incubated at 38 °C. The plates were checked for bacterial growth after 24 h and 48 h. Growing bacteria were identified via colony morphology and growth patterns. Additionally, these quarter milk samples were stored at − 20 °C until the end of the experiment. For analysis, the samples were defrosted and prepared to perform serial dilutions according to Petzl et al. (2016) [49]. Therefore, the quarter milk samples were diluted with 0.9% sterile saline solution. Three degrees of dilution were plated on CSB agar in the case of *S. aureus* challenge and on VRB agar in the case of *E. coli* -challenge. For each degree of dilution, one triplicate was used. The agar plates were incubated for 24 h at 38 °C, and the CFUs were determined by manual colony counting. Evaluable plates contained a minimum of two and a maximum of 300 colonies. Calculation of the colony forming units (CFU/ml) was performed according to Farmiloe et al. (1954) [50]. The value obtained after calculation was multiplied by the factor 10, as only 100 μl were incubated in each degree of dilution.

### Statistical analysis

Data were managed using Microsoft Excel and Access (Microsoft, Redmond, WA, USA). Statistical analyses were performed with GraphPad PRISM 5.04. Power calculation for sample size determination was performed based on results from previously performed IMI models which had indicated higher variability of target parameters during IMI [40, 45]. Two-sample t test power calculation with the target variable SCC in quarter milk samples resulted in the necessity of *n* = 12 per *S. aureus*-group and *n* = 6 per *E. coli*-group. Data were tested for normal distribution via the Shapiro-Wilk test. In the case of normally distributed data, the results are presented as the mean ± standard error of the mean (SEM), in the case of non-normally distributed data or if individuals per subgroup were less than *n* = 7, the results are presented as the median ± interquartile range (IQR). Normally distributed data were compared via unpaired t-tests. If data were not normally distributed or if individuals per subgroup were less than n = 7, the nonparametric Mann-Whitney test was applied. Accordingly, all analyses concerning data originating from the *E. coli* group were performed using the nonparametric Mann-Whitney test, as maximum n = 6 individuals were counted in each group. As described above, uniparous cows in the *S. aureus* group received the pathogen in two udder quarters. Concerning udder secretion and palpatory score as well as SCC and CFU, a mean value was calculated per uniparous cow and used for the statistical analysis and data illustration. Calculated differences with *P* < 0.05 were regarded as significant.

## Supplementary information

**Additional file 1: Table S1.** General health condition scoring scheme. Compromises the general health condition scoring scheme (Score 0–9, modified based on Petzl et al. (2012) [36]), which was applied in the present study to evaluate the severity of the induced mastitis and to detect differences between the divergent haplotypes. The following parameter were scored to evaluate the general health condition of the uniparous cows: heart rate, inner body temperature, filling and activity of the rumen and feed intake.

**Additional file 2: Table S2.** Results of general health condition scoring of Q-/q-uniparous cows after intramammary challenge with *Staphylococcus aureus*. Compromised the results of the applied general health condition scoring of Q-/q-uniparous cows after intramammary challenge with *Staphylococcus aureus*.

**Additional file 3: Table S3.** Results of general health condition scoring of Q-/q-uniparous cows after intramammary challenge with *Escherichia coli.* Compromised the results of the applied general health condition scoring of Q-/q-uniparous cows after intramammary challenge with *Escherichia coli.*

## Data Availability

The datasets used and/or analyzed during the current study are available from the corresponding author on reasonable request.

## Abbreviations

BHI: Brain heart infusion broth
BTA 18: *Bos taurus* autosome
C: Celsius
CFU: Colony forming units
CM: Clinical mastitis
CMT: California mastitis test
CSB: Columbia sheep blood agar
*E. coli*: *Escherichia coli* 1303
FL: Front left quarter
FR: Front right quarter
h: Hour
HL: Hind left quarter
HR: Hind right quarter
IMI: Intramammary infection
IQR: Interquartile range
log: Logartihmized to the base 10
MEC: Mammary epithelial cells
mg: Milligram
ml: Milliliter
p.p.: Postpartum
Q: Favorable haplotype uniparous cow
q: Unfavorable haplotype uniparous cow
QTL: Quantitative trait locus
*S. aureus*: *Staphylococcus aureus* 1027
SCC: Somatic cell count
SCM: Subclinical mastitis
SCS: Somatic cell score
TSB: Tryptic soy broth
VRB: Violet red bile agar
