# Supplementary material for: In vivo model to study the impact of genetic variation on clinical outcome of mastitis in uniparous dairy cows
Source: BMC Vet Res. 2020 Jan 31;16:33. doi: 10.1186/s12917-020-2251-8 (PMC6995066; doi:10.1186/s12917-020-2251-8)
Supplement: Supplementary file 1 — Additional file 1: Table S1. General health condition scoring scheme. Compromises the general health condition scoring scheme (Score 0–9, modified based on Petzl et al. (2012) [36]), which was applied in the present study to evaluate the severity of the induced mastitis and to detect differences between the divergent haplotypes. The following parameter were scored to evaluate the general health condition of the uniparous cows: heart rate, inner body temperature, filling and activity of the rumen and feed intake. [file 12917_2020_2251_MOESM1_ESM.docx]

## Additional file 1: Table S1: General health condition scoring scheme

| Parameter | Score |
| --- | --- |
| Intravaginal temperature (°C) |  |
| 37.5 – 39.4 | 0 |
| 39.5 – 40.0 | 1 |
| 40.1 – 41.4 | 2 |
| ≥ 41.5 or < 37.5 | 3 |
|  |  |
| Heart rate (beats/min) |  |
| < 90 | 0 |
| ≥ 90 | 1 |
|  |  |
|  |  |
| Rumen contraction (contractions/2 min) |  |
| +++ | 0 |
| ++(+) | 0.5 |
| ++- | 1 |
| +(+)- | 1.5 |
| +-- | 2 |
| (+)-- | 2.5 |
| --- | 3 |
|  |  |
| Feed intake |  |
| good | 0 |
| good - moderate | 0.5 |
| moderate | 1 |
| moderate - none | 1.5 |
| none | 2 |
|  |  |

**Severity of disease is classified by ranges of a total systemic clinical score: healthy (0-3), mild disease (4-5), moderate disease (6-7), severe disease (8-9).**
