# Supplementary material for: In vivo model to study the impact of genetic variation on clinical outcome of mastitis in uniparous dairy cows
Source: BMC Vet Res. 2020 Jan 31;16:33. doi: 10.1186/s12917-020-2251-8 (PMC6995066; doi:10.1186/s12917-020-2251-8)
Supplement: Supplementary file 2 — Additional file 2: Table S2. Results of general health condition scoring of Q-/q-uniparous cows after intramammary challenge with Staphylococcus aureus. Compromised the results of the applied general health condition scoring of Q-/q-uniparous cows after intramammary challenge with Staphylococcus aureus. [file 12917_2020_2251_MOESM2_ESM.docx]

## Additional file 2: Table S2: Results of systemic health condition scoring of Q-/q-uniparous cows after intramammary challenge with *Staphylococcus aureus*.

| time (h) relative to *Staphylococcus aureus* challenge | Median ± IQR (Score) | | P-value |
| --- | --- | --- | --- |
|  | **GQ (n = 12)** | **kq (n = 12)** |  |
| 0 | 1.00 ± 0.00 | 1.00 ± 0.00 | P = 0.68 |
| 12 | 1.00 ± 0.50 | 1.00 ± 1.00 | P = 0.97 |
| 24 | 1.50 ± 1.50 | 1.25 ± 1.38 | P = 0.86 |
| 36 | 1.50 ± 2.00 | 1.75 ± 2.00 | P = 0.72 |
| 48 | 1.00 ± 2.13 | 1.50 ± 0.50 | P = 0.90 |
| 60 | 1.25 ± 1.75 | 1.25 ± 1.63 | P = 0.88 |
| 72 | 1.25 ± 2.13 | 1.00 ± 0.88 | P = 0.52 |
| 84 | 1.50 ± 1.75 | 1.25 ± 0.88 | P = 0.69 |
| 96 | 1.00 ± 0.00 | 1.00 ± 0.00 | P = 0.42 |

*IQR = Interquartile range*
