# Supplementary material for: In vivo model to study the impact of genetic variation on clinical outcome of mastitis in uniparous dairy cows
Source: BMC Vet Res. 2020 Jan 31;16:33. doi: 10.1186/s12917-020-2251-8 (PMC6995066; doi:10.1186/s12917-020-2251-8)
Supplement: Supplementary file 3 — Additional file 3: Table S3. Results of general health condition scoring of Q-/q-uniparous cows after intramammary challenge with Escherichia coli. Compromised the results of the applied general health condition scoring of Q-/q-uniparous cows after intramammary challenge with Escherichia coli. [file 12917_2020_2251_MOESM3_ESM.docx]

## Additional file 3: Table S3: Results of systemic health condition scoring of Q-/q-uniparous cows after intramammary challenge with *Escherichia coli.*

| time (h) relative to *Escherichia coli* challenge | Median ± IQR (Score) | | P-value |
| --- | --- | --- | --- |
|  | **GQ (n = 6)** | **kq (n = 6)** |  |
| 0 | 1.00 ± 0.88 | 1.00 ± 0.13 | P = 0.92 |
| 12 | 1.50 ± 2.88 | 1.75 ± 2.25 | P = 1.00 |
| 24 | 1.00 ± 1.50 | 1.00 ± 0.25 | P = 0.44 |

*IQR = Interquartile range*
